# E-hooks provide guidance and a soft landing for the microtubule binding domain of dynein

**DOI:** 10.1038/s41598-018-31480-9

**Published:** 2018-09-05

**Authors:** Nayere Tajielyato, Lin Li, Yunhui Peng, Joshua Alper, Emil Alexov

**Affiliations:** 1 Department of Physics and Astronomy, Clemson University, Clemson, SC 29634 USA; 2 Department of Physics, University of Texas at El Paso, El Paso, TX 79912 USA

## Abstract

Macromolecular binding is a complex process that involves sensing and approaching the binding partner, adopting the proper orientation, and performing the physical binding. We computationally investigated the role of E-hooks, which are intrinsically disordered regions (IDRs) at the C-terminus of tubulin, on dynein microtubule binding domain (MTBD) binding to the microtubule as a function of the distance between the MTBD and its binding site on the microtubule. Our results demonstrated that the contacts between E-hooks and the MTBD are dynamical; multiple negatively charted patches of amino acids on the E-hooks grab and release the same positively charged patches on the MTBD as it approaches the microtubule. Even when the distance between the MTBD and the microtubule was greater than the E-hook length, the E-hooks sensed and guided MTBD via long-range electrostatic interactions in our simulations. Moreover, we found that E-hooks exerted electrostatic forces on the MTBD that were distance dependent; the force pulls the MTBD toward the microtubule at long distances but opposes binding at short distances. This mechanism provides a “soft-landing” for the MTBD as it binds to the microtubule. Finally, our analysis of the conformational states of E-hooks in presence and absence of the MTBD indicates that the binding process is a mixture of the induced-fit and lock-and-key macromolecular binding hypotheses. Overall, this novel binding mechanism is termed “guided-soft-binding” and could have broad-reaching impacts on the understanding of how IDRs dock to structured proteins.

## Introduction

Cells regulate the motility of cytoskeletal motor proteins for various biological functions^1–4^. A vast network of mechanisms directly regulate the motility of cytoskeletal motor proteins, including autoinhibition^5^, direct phosphorylation^6,7^, motor associated proteins^8–12^, cargo binding^13,14^, and small molecules and ions^15,16^, to name a few. Moreover, cytoskeletal motor motility along filamentous tracks is a protein-protein interaction between the motor and the filament. Therefore, motility regulation mechanisms not only are targeted to functions of the motor itself, but also affect interactions with their tracks. Microtubule motor proteins, for example, can change their motile properties in response to signals written in the “tubulin code”^17^ based on the specific tubulin isoforms from which microtubules are assembled^18^, and post translational modifications (PTMs) to those microtubules^19^. α-tubulin lysine 40 acetylation, which is a prominent PTM found in the lumen of stable microtubules^20,21^, regulates the motility of kinesin and dynein motor proteins both *in vivo* and *in vitro*^22,23^. The other microtubule PTMs that regulate motor motility, i.e. detyrosination, polyglutamylation and polyglycylation^24^, predominantly occur on the C-terminal tails of tubulin, hereafter called E-hooks.

E-hooks are highly negatively charged, intrinsically disordered regions (IDR)^25^ at the C-terminus of both α- and β-tubulin^26^. E-hooks decorate the outside of the microtubule lattice, interact with many microtubule-associated proteins^25^. Much of the difference between the various isoforms of both α-tubulin and β-tubulin is due to primary amino acid sequence divergence and posttranslational modifications localized to E-hooks^19^. Cells regulate many processes through E-hook related mechanisms, including microtubule dynamics^26,27^, end binding protein recognition of microtubule plus ends^28^, spastin severing of microtubules^29^, microtubule motor protein motility^30,31^ and force production^32^, kinetochore attachment and diffusion along the mitotic spindle^33,34^, tau- microtubule interactions^35^, and multiple other processes.

The motility of the dynein family of microtubule motor proteins is highly regulated, in part because there is only one cytoplasmic dynein to perform the litany of microtubule minus-end directed motor functions while there are many kinesin family members to perform the plus-end directed functions^3^. E-hooks have been implicated in the regulation of cytoplasmic dynein’s processivity^36,37^,and speed^30^. Additionally, E-hooks have been implicated in the regulation of axonemal dynein’s speed and processivity *in vitro*^23^, and the polyglutamylation of E-hooks localized to particular protofilaments on axonemal microtubule tunes the beat of flagellar motility^38,39^. Recently, we showed that changes in the processivity and speed of cytoplasmic dynein associated with certain mutations^40^ may be due to how long-range electrostatic interactions affect the binding of dynein’s microtubule binding domain (MTBD) to the microtubule^41^. However, this analysis was done in the absence of E-hooks because, in part, structure of E-hooks in the presence or absence of the MTBD has not been solved experimentally. Here we investigate the role of E-hooks on MTBD-microtubule binding, and we use the results to introduce a novel protein-protein binding mechanism.

Macromolecular binding is critical to molecular assemblage formation^42^, signal transduction^43^, allosteric regulation^44^, molecular reactions^45^ and transport^46^. The essential processes of macromolecular binding include binding partner recognition, receptor and ligand pre-binding orientation, and finally physical docking, during which the receptor and the ligand undergo conformational changes. If the conformational changes are small, then the corresponding binding mechanism is “lock-and-key”^47,48^. If the conformational changes are large, then the binding mechanism is an “induced-fit”^48^. When the induced-fit binding mechanism involves intrinsically disordered proteins (IDPs) or regions (IDRs)^49^, they become structured^50^ by shifting the occupancy of distinct bound and unbound conformational states from a highly populated unbound state to a highly populated bound state^51,52^.

Here we use molecular dynamics (MD) simulations to investigate the role of tubulin E-hooks in MTBD-microtubule binding. We identify key residues on the E-hooks and MTBD that make contacts as the MTBD approaches the microtubule and binds to it. We also test the hypothesis that the role of E-hooks in MTBD-microtubule binding is consistent with an “induced-fit” mechanism. Furthermore, we compute the electrostatic force that E-hooks exert on the MTBD as it approaches microtubule. Our results provide details on specific MTBD-E-hook interactions and binding forces, and we describe a novel binding mechanism that is different from lock-and-key and induced-fit called “guided-soft-binding”.

## Results

We investigated the role that the α-tubulin and β-tubulin E-hooks play in the docking of cytoplasmic dynein’s MTBD to a microtubule. To do so, we analyzed the E-hook-MTBD contacts at increasing MTBD-microtubule distances, the conformational changes of the E-hooks as the distance increases, the role of individual amino acids within the MTBD and E-hooks, and the electrostatic forces acting between E-hooks and MTBD.

### The number of E-hook-MTBD contacts vary with MTBD-microtubule distance and simulation time

**Figure 1:**
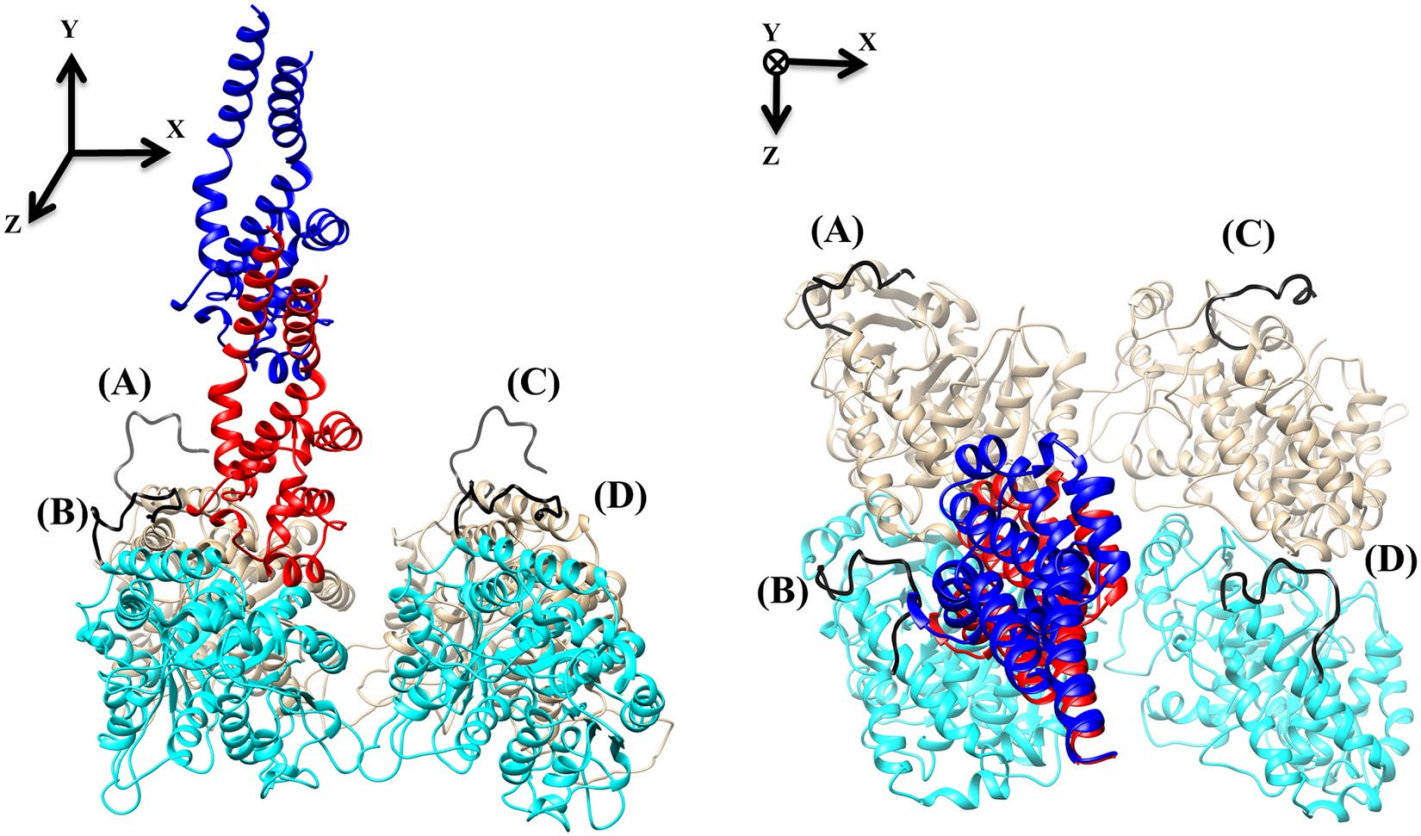
The MTBD-microtubule structure. The side view (left) and top view (right) of two tubulin dimers and a MTBD in docked position (red) and at a distance of 35 Å (blue). In our structure, we refer to the E-hooks as chains (**A**–**D**) where (**B** and **D**) are β-tubulin (cyan) E-hooks, and (**A** and **C**) the corresponding α-tubulin (brown) E-hooks. All four E-hooks present in the structure are colored black and labeled according to the chain letter of the corresponding tubulins.

We investigated how the number of contacts made between the E-hooks on a microtubule segment and the MTBD (Fig. 1) varies with MTBD-microtubule distance by offsetting the MTBD perpendicularly from its bound position by 5, 15, 25, 35, 45, and 55 Å. In our structural model of the microtubule segment, only the β-tubulin E-hooks (labeled as B and D, Fig. 1) made contacts with the MTBD, therefore we restrict the presentation of the results to those associated with these E-hooks.

**Figure 2:**
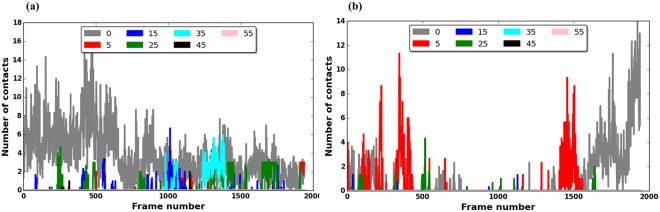
The number of E-hook-MTBD contacts vary with MTBD-microtubule distance and simulation time. (**a**) The average number of E-hook B-MTBD contacts at various distances. (**b**) The average number of E-hook D-MTBD contacts at various distances. In both panels, the number of contacts in each frame is calculated by counting the number of E-hook-MTBD contacts in a given frame and averaging over three MD trajectories (see Methods), the bound position (gray plot) corresponds to 0 Å, and the MTBD-microtubule distances of 5, 15, 25, 45, 55 Å are plotted as indicated in the legend. Note that only the snap shots of the last 10 ns of a total 20 ns simulation time are plotted.

First, we found that the MTBD made many more contacts with E-hook B than E-hook D (Fig. 2), regardless of MTBD-microtubule distance. This was due to the distance between the center of mass of the MTBD and the base of E-hook being smaller for E-hook B (47.12 Å) than for E-hook D (75.58 Å), as calculated from the 3D structure.

Second, we found that the number of MTBD-E-hook contacts fluctuated over the simulation time at all distances, with no obvious pattern. At some distances, the E-hooks made more contacts in the beginning of a run, and at other distances this happened at the middle or at the end of the run (Fig. 2). Particularly for large distances, we found frequent “spikes” of contacts and then long simulation times without any contacts (Fig. 2). This indicates the dynamical nature of MTBD-E-hooks interactions and suggests that the E-hooks do not bind permanently to the MTBD, rather they “grasp” MTBD for a short period of time and then adopt an unbound conformation.

Additionally, we found that ability of the E-hooks to make contact with the MTBD as a function of distance was different for E-hooks B and D (Fig. 2). The maximum number of contacts between E-hook D and the MTBD occurred at distances of 0 and 5 Å, and we found no contacts for distances greater than 25 Å (Fig. 2b). In contrast, E-hook B made more contacts with the MTBD at both the docked position (distance of zero) and at intermediate distances of 25 and 35 Å than either close (5 and 15 Å) or far (45 and 55 Å) distances (Fig. 2a). However, we found that E-hook B made a few contacts with the MTBD even at a distance of 45 Å (Fig. 2a).

We observed similar trends in the number of MTBD-E-hook contacts as a function of time among the three runs for most of cases. However, there were occasional MD simulations in which one of the E-hooks remained bound to the MTBD for long stretches of simulation time. One such run occurred at a distance of 15 Å in which we found that E-hook chain D remained bound for the entire simulation run (see Supplementary Information, Fig. S1). Because this case was an outlier, we did not include it in the analysis. The existence of such outlying runs, however uncommon, suggests that E-hook interactions with the MTBD may involve conformational changes that require significant sampling. However, even in the outlying runs, the overall pattern of frequent “spikes” in the number of contacts is preserved with the only difference being the absolute number of contacts.

### The MTBD traveled along the length of the E-hooks as it approached the microtubule

**Figure 3:**
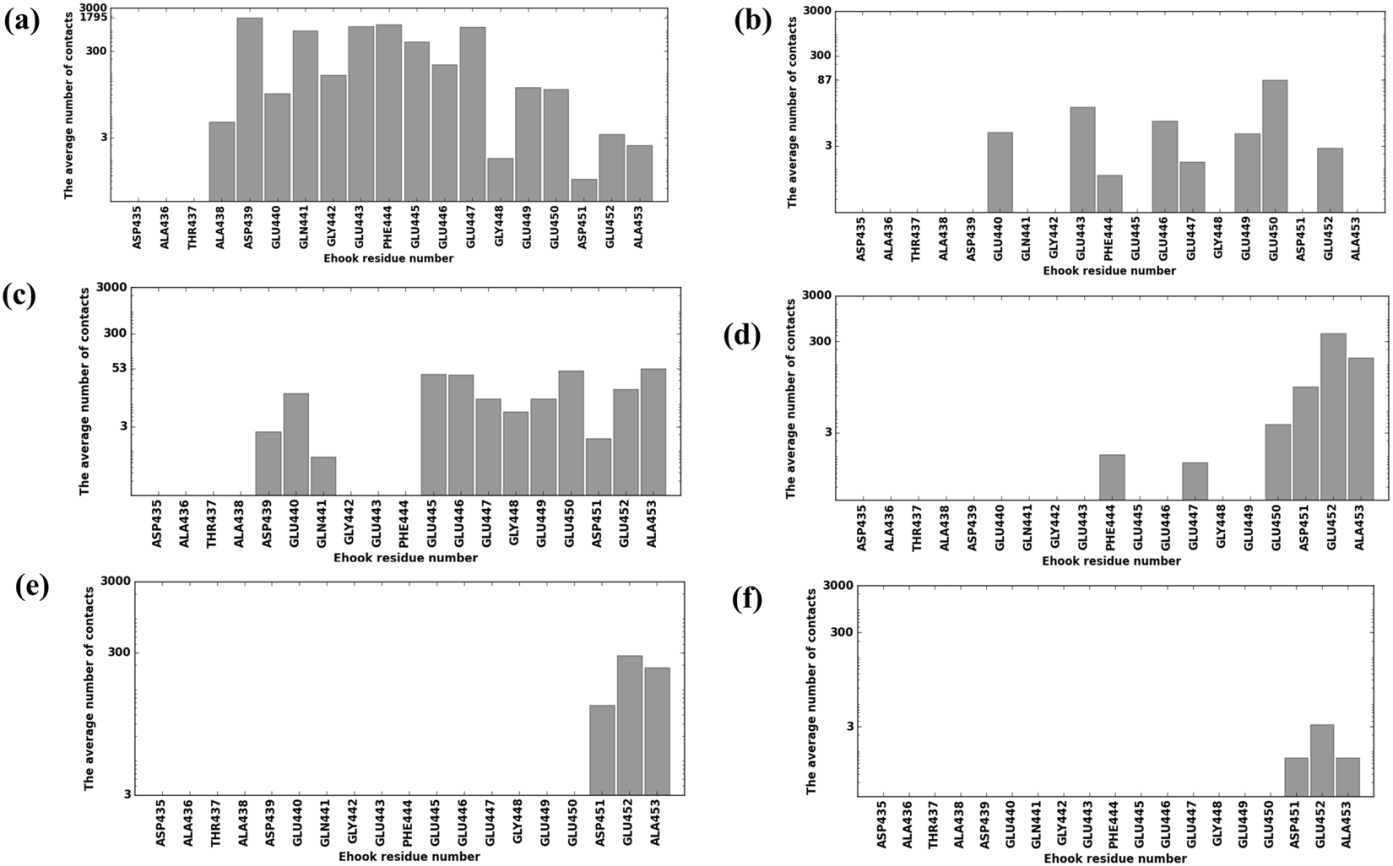
The MTBD made contacts (in logarithmic scale) along the length of E-hook B as the MTBD approached the microtubule. Bars represent the average number of contacts made by each E-hook B residue and the MTBD at MTBD-microtubule distances of 0, 5, 15, 25, 35, and 45 Å (panels a–f, respectively). Three independent runs (see Methods) were analyzed and results averaged for each distance, and 2000 total frames were analyzed for each run.

**Figure 4:**
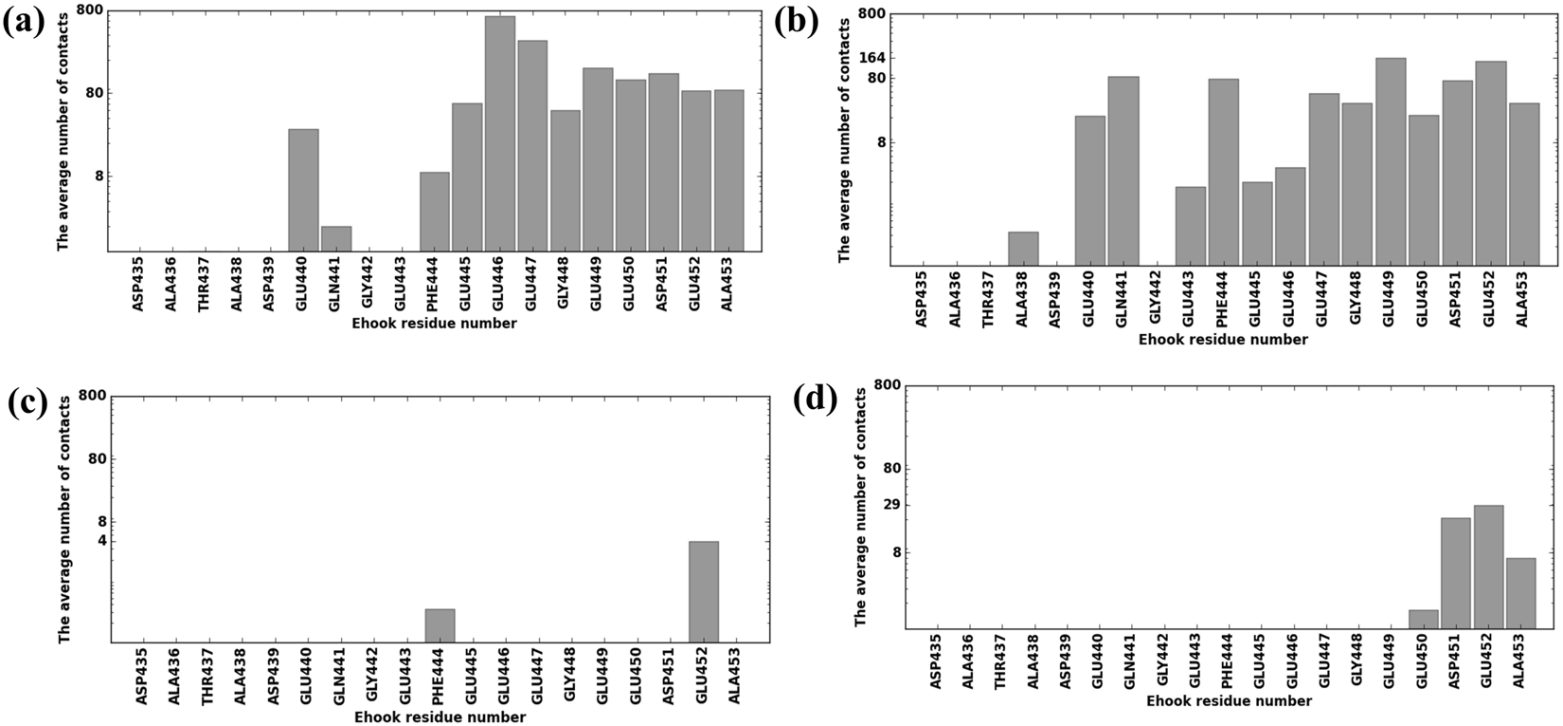
The MTBD made contacts primarily with the distal tip of E-hook D. Bars represent the average contacts made by each E-hook D residue (in logarithmic scale) and the MTBD at MTBD-microtubule distances of 0, 5, 15 and 25 Å (panels a–d, respectively). Three independent runs (see Methods) were analyzed and results averaged for each distance, and 2000 total frames were analyzed for each run.

To understand the effects of possible mutations^53^ and their plausible implications on disease^54,55^ we identified which E-hook residues made contacts with which MTBD residues as a function of MTBD-microtubule distance. We tabulated (Table S1) and plotted the number of contacts each E-hook residue made with the MTBD (Figs 3 and 4), and we found that, while almost all of the residues made some contacts with the MTBD at some distance, most of the contacts were made by residues situated in the middle of E-hook B, including Asp439, Glu443, Phe444 and Glu447, when the MTBD was at the binding position (distance = 0, Fig. 3a). At the largest MTBD-microtubule distance, most of the contacts were made by residues at the distal end of the E-hook (Asp451, Glu452, and the backbone of Ala 453, Fig. 3f). Between these two extrema (Fig. 3a and f), the MTBD-E-hook B contacts tended to move distally along the E-hook as the MTBD-microtubule distance was increased (Fig. 3a–f).

The situation was quite different for E-hook D (Fig. 4). We found that residues at the proximal end of the E-hook did not make contacts with the MTBD, even at the bound position (Fig. 4a), likely because the distance from the MTBD to E-hook D was larger it was to E-hook B (see Methods), preventing the proximal E-hook D residues from reaching the MTBD. We also noted that E-hook D made almost no contacts with the MTBD at a distance of 15 Å (Fig. 4c), while it managed to establish many at distance of 25 Å. We observed no MTBD-E-hook D contacts at distances larger than 25 Å (Fig. 4d).

**Figure 5:**
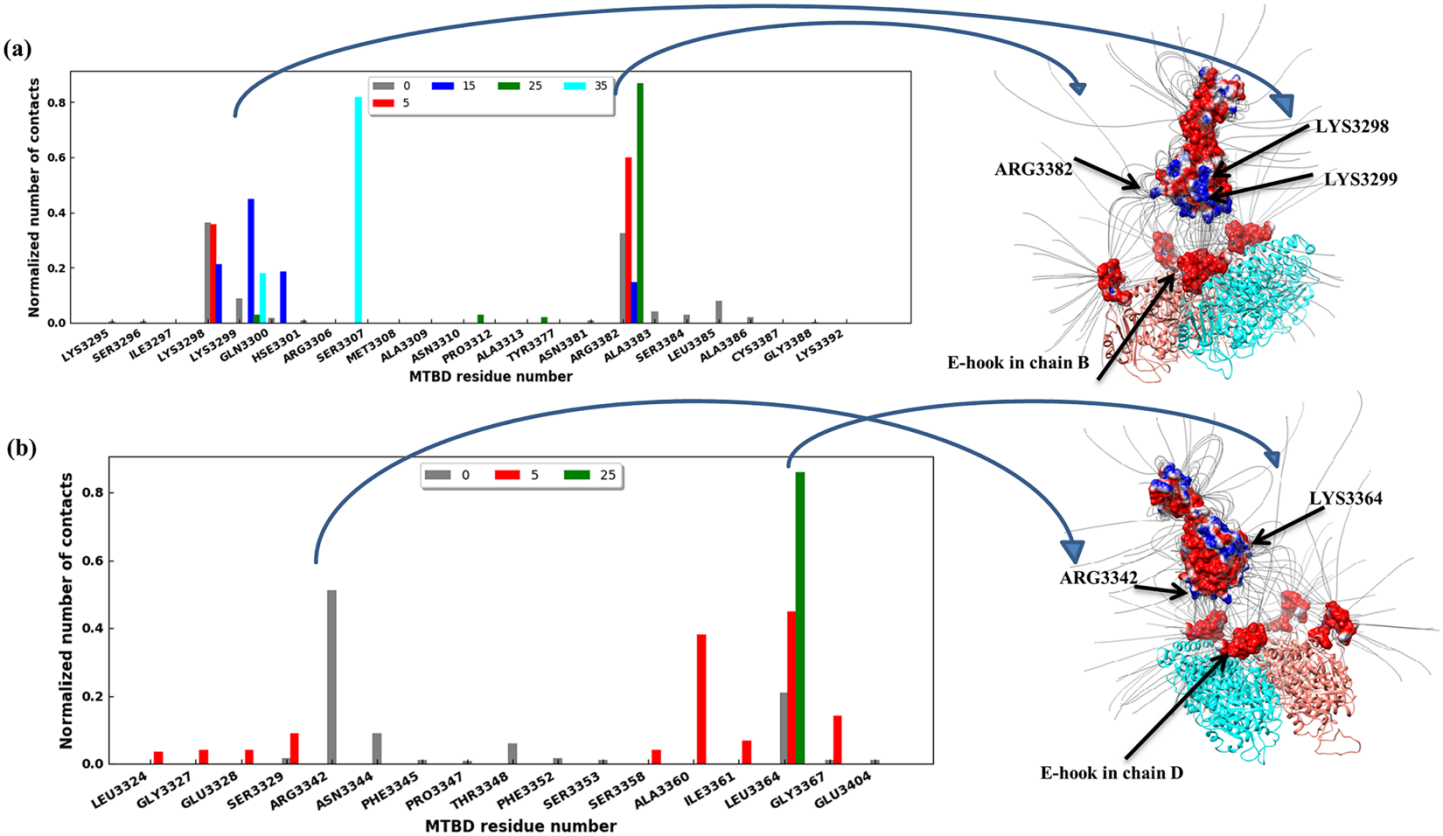
Certain MTBD residues made most of the contacts with E-hooks. (**a**) Histogram of the number of E-hook B contacts each residue of the MTBD makes constants at various MTBD-microtubule distances (see legend). (**b**) Histogram of the number of E-hook D contacts each residue of the MTBD makes constants at various MTBD-microtubule distances (see legend). In each panel, the number of contacts was normalized by the total number of contacts each particular distance over the 3 independent runs of 2000 total frames each (see Methods). Note that no results are shown here for distances larger than 35 Å because there are few contacts, however these numbers are provided in supplementary material. The right panels show the MTBD at distance = 15 Å with electrostatic potential mapped onto its surface, and the α-/β- tubulin dimer with A, B, C, D chains E-hooks highlighted (red). Note, a frame was chosen in which the E-hooks are shown not making contact with the MTBD for clarity. The residues of MTBD making most of the contacts with E-hooks are labeled.

Similarly, we identified key residues within MTBD by tabulating (Table S2) and plotting the number of contacts each MTBD residue made with E-hook B (Fig. 5a) and E-hook D (Fig. 5b) at various distances, normalized by the total number of contacts at each distance, respectively. We found that Lys3298 and Arg3382 were the residues on the MTBD that made the most contacts with E-hook-B at a MTBD-microtubule distance of 0 Å, accounting for more than 69% of the contacts (Fig. 5a). As the distance was increased, other residues got involved as well, including Arg3306 and Lys3299 (Fig. 5a), indicating that E-hook B interacted with slightly different surface patches of the MTBD at different distances. E-hook D interacted with an entirely different set of residues (Fig. 5b), most prominently Arg3342, which made 51% of the contacts when the MTBD-microtubule distance is 0 Å (bound state for E-hook D). As the distance increased, Lys3364 and Lys3367 took over, and made most of the contacts (Fig. 5b).

Three MTBD helices (H1, H3, and H6, Fig. S2)^56^ contain a high density of conserved, surface-accessible residues, and mutation of several of these conserved residues significantly affected MTBD binding to microtubule^56^. Here, we found that these same H1, H3, and H6 helices were the regions on the MTBD that had the most contacts with the E-hooks as the MTBD approached the microtubule (Table S3). Moreover, the conformational changes, as quantified by the average RMSD, of these three helices were larger than for the other MTBD helices (Table S3). We noted that the magnitude of the conformational changes correlated with the number of MTBD-E-hook contacts.

### E-hook conformations and cluster analysis

Protein-protein binding induces conformational changes in the binding partners^57–59^. The conformational changes associated with transitioning between the unbound and bound state are thought to reflect a change in the population of conformational states that exist within the ensemble of states when the proteins are isolated, rather than the generation of entirely new conformational states^60^. In other words, conformational states that were highly populated in the ensemble of states of the isolated structures are sampled with much lower frequency upon binding, and conformational states that exist but are very lowly populated in the ensemble of states of the isolated structures become sampled with much higher frequency upon binding.

To determine whether MTBD-E-hook interactions induce a drop in the population of E-hook conformational states that are highly populated in the absence of the MTBD and an increase in the population of conformational states that lowly populated in the absence of the MTBD, we carried out a clustering procedure of the conformational states that are found in our MD simulations of both free E-hooks and the E-hooks with the MTBD bound situated at various distances. To be consistent in comparing the conformational states of unbound and bound cases, we produced a representative structure for the first five most populated clusters in each case (Table S4). Figure S4 shows the representatives and the corresponding structural files in PDB formal and relevant movies are available for download from http://compbio.clemson.edu/downloads. We found that cluster 1 and 2 were occupied in about 53% the calculated free E-hook B conformational states (Table S4, column 1 of all sub-tables), and clusters 3, 4, and 5 accounted for the next 32% of the snapshots (Table S4, column 1 of all sub-tables). The remaining 15% did not fall into any of the identified clusters. Similarly, clustering was done for the bound state (Table S4) and at the various MTBD-microtubule distances studied in this work. For each E-hook in isolation/E-hook in the presence of the MTBD cluster pair, we calculated the RMSD between the representative structures (see Methods, values reported in Table S4). A small RMSD value indicated that the E-hook cluster in presence of the MTBD, either bound or situated at a particular distance, was similar to the representative of the E-hook conformational state cluster when isolated from the MTBD. Conversely, a large RMSD value indicated that the E-hook cluster in presence of MTBD was quite different from the representative of the E-hook conformational state cluster when isolated from the MTBD.

**Figure 6:**
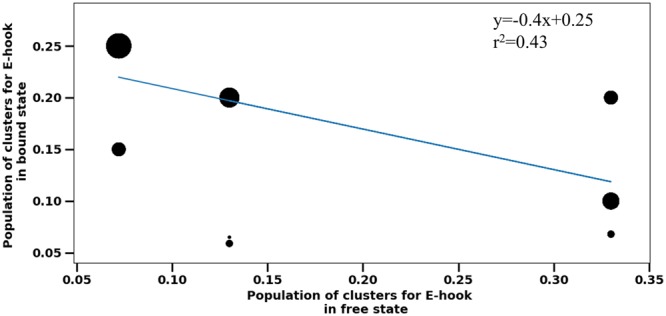
The correspondence between the free clusters and clusters in bound state for clusters having more than 10 contacts (different thresholds were tested, but the results remain qualitatively the same). Each data point represents the corresponding occupancies listed in Table S4. The datapoints size is proportional to the number of contacts, i.e. large circles corresponding to datapoints with many contacts.

We found that there was a tendency for clusters having a large number of MTBD-E-hook contacts and a large occupancy in presence of the MTBD to be structurally similar, as indicated by a small RMSD, to clusters seen in free state but with low probability (occupancy). This indicates that bound conformations were indeed present in the unbound state, but with low probability. We plotted the population of E-hook structural clusters in the free state vs the bound state and performed a linear regression with the data weighted by the average number of MTBD-E-hook B contacts, Nc, found in the 2000 frame MD simulation (Fig. 6). The tendency observed in the data (Table S4) was confirmed by the regression, however, the R^2^ was only 0.43, indicating that this tendency was weak.

We also found that the clusters making very few contacts did not show the same tendency as those that do (Fig. S3). However, even without a clear tendency, we did find that clusters not making contact with the MTBD in any frame (N_c_ = 0) were influenced by the presence of MTBD. In particular, the RMSD values indicated that the highly and lowly populated clusters of free E-hook structures were not similar to the equally populated clusters in the presence of MTBD, even if they never made contact with the MTBD. This was even true for clusters at distances larger than 45 Å, in which the E-hook cannot reach the MTBD. These results indicate that the E-hook’s confirmation is affected by the MTBD, possibly via long range electrostatic interactions. Similar tendency was found comparing the secondary structure elements (SSE) of E-hooks (see Fig. S5). However, note that these observations should be taken with caution because the cluster analysis accounted for only about 60% of snap shots. The other 40% of snap shots that were not clustered perhaps indicate the dynamic nature of E-hook interactions with MTBD.

### Electrostatic force between MTBD and E-hook

To investigate the role of electrostatics in the binding of the MTBD to the microtubule in presence of E-hooks, we first calculated the pKa of the titratable residues using DelPhi-pKa^61^. It was found that most of titratable residues are fully ionized at pH = 7.0, as described in our previous work^41^, and we found that all the glutamic acid and aspartic acid residues within the E-hooks, specifically, were fully ionized at pH 7.0 when E-hooks were unbound to MTBD (Note that calculating pKa values for all snap shots and all distances is computationally prohibited). Thus, in the modeling below all glutamic acid and aspartic acid residues within E-hooks were kept charged.

**Figure 7:**
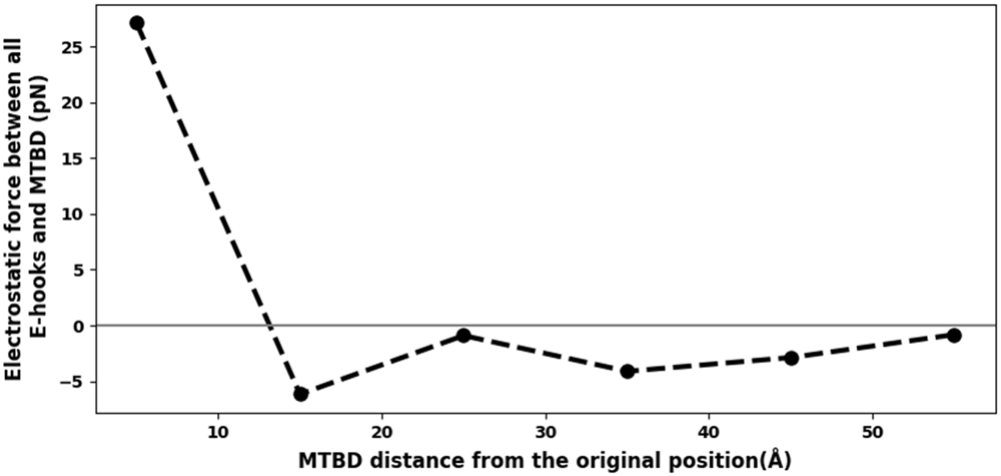
Perpendicular component electrostatic force on the MTBD due to the E-hooks reels in and provides a soft landing for the MTBD. The electrostatic force component perpendicular to microtubule interface (the y direction, Fig. S1) plotted as a function of MTBD-microtubule distance. Note that the force for d = 0 Å was omitted from this analysis to avoid excessively large forces resulting from atoms positioned artificially too close to each other in the computation. Also note that the electrostatic force between MTBD and E-hooks was calculated by DelphiForce using all snap shots at each distance, and then the average value is reported here.

We calculated the electrostatic fields (Fig. 5 at distance = 15 Å, for example) using Delphi and corresponding forces using DephiForce between the MTBD and E-hooks at each MTBD-microtubule distance. We found that there were strong interactions between the E-hooks and charged patches of the MTBD, even for cases in which the E-hook did not make direct contact with the MTBD (Fig. 5). This result demonstrates that long-range electrostatic interactions between the E-hooks and the MTBD affect the MTBD as it approaches the microtubule. To further quantify the role of electrostatics on the binding, we plotted the magnitude of the perpendicular component of electrostatic force acting on the MTBD due to the all four E-hooks (Fig. 1 for E-hook positions) as a function of MTBD-microtubule distance (Fig. 7). We found that the electrostatic force pulled the MTBD domain toward the microtubule, as indicated by negative values of force, at large distances. The electrostatic forces thus “reeled” the MTBD into its binding position. However, as the MTBD made its final approach to the binding position, the electrostatic forces originating from the E-hooks opposed the binding, as indicated by positive force values. Thus, the effect of the E-hooks is to slow the approach of the MTBD as it finally docks, providing a “soft” landing onto the microtubule for the MTBD.

## Discussion

The results presented in this work show that microtubule E-hooks play a dual role in cytoplasmic dynein MTBD binding to the microtubule that we call “guided-soft-binding”. On one hand the E-hooks guide the MTBD towards the binding position via direct and long-range electrostatic interactions, and on the other, E-hooks provide a force that opposes the binding as the MTBD makes physical contact. More generally, the results suggest a novel mixed binding model, in which an intrinsically disordered domain, the E-hook in this case, is docked to its binding partner, the MTBD in this case, through a mixture of the induced-fit and lock-and-key macromolecular binding hypotheses.

Our simulations indicate that the β-tubulin E-hooks on neighboring protofilament within the microtubule (termed the B and D E-hooks, Fig. 1) act as a team to reel the MTBD into the microtubule binding site and then provide a soft landing as the MTBD finally binds. Even well before making first contact, the E-hooks exerts a force that pulls the MTBD toward its binding site (Fig. 7). As the MTBD comes into a range that the E-hooks can make contact, the MTBD is guided to its docking position on the microtubule by a processive movement from the distal end to the base of the B E-hook (Fig. 3), all the while being stabilized by only few interactions with the D E-hook (Fig. 4). This movement down the E-hook is enabled by interactions between the same positively charge patches on the MTBD (Fig. 5) and multiple patch of negatively charged amino acids distributed along the E-hooks (Figs 3 and 4). However, at a MTBD-microtubule distance of 5 Å, just before reaching the MTBD reaches its final docking position, we found that the MTBD is passed from the B E-hook to the D E-hook; the number of contacts with the B E-hook drop significantly at this distance (Fig. 3), but this is compensated by an increase in the number of D E-hook contacts (Fig. 4). However, in its final docking position (distance = 0 Å), the D E-hook makes many contacts with the MTBD again (Fig. 4). Thus, the MTBD is passed from one β-tubulin E-hook to the neighboring β-tubulin’s E-hook as it is reeled into its biding site.

Our results may help to explain multiple experimentally observed phenomena. The ability of β-tubulin E-hooks to reel in the MTBD may explain how cytoplasmic dynein exhibits an approximately 2-fold increased processivity, on microtubules with both α- and β-tubulin E-hooks and those with only β-tubulin E-hooks as compared to those with only α-tubulin E-hooks and those that lack E-hooks entirely^30,36^. Additionally, our results provide insight into the mechanism that causes cytoplasmic dynein exhibits moves slightly slower on microtubules that have β-tubulin E-hooks than those that do not^30^. Our results could be further explored experimentally in motility assays or optical tweezers assays comparing results between wildtype structures and those with key tubulin (e.g. E446, E449, D450, E452) and MTBD (e.g. K3298, K3299, R3342, K3336, and R3382) residues mutated to those with non-polar amino acids, i.e. alanine (see also Table S5 for calculated binding free energy changes upon mutating these residues). With high enough resolution optical tweezers, direct comparison to Fig. 7 should be possible.

Also, we found that the E-hooks provide soft landing for the MTBD on the microtubule. The contribution of E-hooks to the net force exerted on the MTBD by the microtubules is in the positive direction (tends to push the MTBD apart from the microtubule, Fig. 7) at distances of 0 and 5 Å. This pushing force will slow the MTBD down at the final approach, which is dominated by electrostatic interactions between the MTBD and non-E-hook residues on the microtubule that pull the MTBD to its final binding site^41^. To the best of our knowledge, the dual-role binding mechanism that we call “guided-soft-binding,” which provides pulling forces to reel it in a ligand at long range and provide pushing forces to cause a soft landing at close range, has never been described in the literature. Our work raises the possibility that this is not only a unique role of E-hooks in the MTBD binding to microtubule, but also it might be a feature of IDR-mediated protein-protein binding more broadly.

Finally, our investigation revealed another distinctively novel feature of MTBD binding to microtubule in presence of E-hooks that was not reported elsewhere for macromolecular binding. The analysis of the conformational states of free E-hooks and E-hooks in presence of MTBD (Table S4 and Fig. 6) showed that the binding does not follow either the lock-and-key or the induced-fit mechanisms. We speculate that this is due to the dominance of long-range electrostatic interactions that provide weak coupling even at large distance between MTBD and microtubule. Thus, the E-hooks still interact with the MTBD even though they not make physical contacts to it, and this does not require adopting particular conformation states. Perhaps this explains our results in which we do not see the typical for IDR scenario when the binding makes the IDR ordered, simply because most of the MTBD-E-hook interactions do not involve the formation of direct contacts.

In all, our results indicate novel roles of tubulin’s E-hooks on the binding of cytoplasmic dynein’s MTBD to the microtubule. Because the E-hook is known to regulate many other microtubule associated protein-microtubule interactions, our data suggest that similar computational studies could reveal multiple novel protein-protein binding mechanisms in these cases. Such knowledge could have broad reaching implications on biological functionality driven by the microtubule cytoskeleton. Even beyond E-hooks, the phenomena revealed here could be fundamental to many IDR or IDP – structured protein binding mechanisms.

## Methods

The 3D structure preparation of microtubule segment and MTBD is described in supplementary material.

The 3D structure of E-hooks from neither α-tubulin nor β-tubulin is available experimentally. Therefore, the structures were generated in silico by Profix (http://wiki.c2b2.columbia.edu/honiglab_public/index.php/Software:Jackal_General_Description)^62^ using the sequence α-tubulin (VGVDSVEGEGEEEGEEY) and β-tubulin (DATADEQGEFEEEGEEDEA) of *Bos taurus*.

To investigate the interactions between the microtubule, including the E-hooks, and the MTBD at various distances, the MTBD was displaced from the experimentally determined bound position (source code: 3J1T.pdb^40^) along the axis perpendicular to the microtubule (the y-axis in Fig. 1) by 5, 15, 25, 35, 45, and 55 Å.

### Molecular dynamics (MD) simulations

The MD simulations were done with NAMD^63^ details are provided in supplementary material.

The MD simulations were run independently three times for each structure with different starting atomic velocities. Each simulation was carried out for a total of 20 ns, and the last 10 ns of the resulting trajectories were analyzed by VMD^64^.

### Analysis of contacts

To analyze the interaction between the MTBD and each E-hook, all atoms, except the hydrogen atoms, were considered. If a heavy atom of E-hook was within 4 Å of an atom of MTBD, this was counted as a contact. The contacts were analyzed using VMD. In the Results section, the contact number is averaged over three independent MD trajectories.

### Analysis of conformational states

To investigate how the E-hook conformations change as a function of MTBD-microtubule distance, snap shots of the tubulin structures with the hydrogen atoms removed were clustered using the “cluster” tools in gromacs^65,66^. Cluster analysis was performed by the Daura algorithm^67^ using a C-alpha root-mean-square deviation (RMSD) cutoff of 1.5 Å. The cluster analysis is based on RMSD between conformations. The first five populated clusters were analyzed in our study. The most common conformation in the pool was selected as the representative structure of each cluster. Note that the entire structure of the corresponding tubulin was used during clustering, instead of just the E-hook, to account for the orientation of the E-hook with respect to the microtubule.

Furthermore, an analysis of the conformational states of the E-hooks both in the absence (unbound or free state) and presence of the MTBD (bound state) was carried out. The correspondence of clusters in the unbound and bound states was inferred using the RMSD of their representative structures. Thus, two clusters were deemed to be similar if the RMSD between their representatives was the smallest one in the list.

### Electrostatic potential and force

The 3D spatial distribution of electrostatic potential was calculated with DelPhi, which numerically solves the Poisson-Boltzmann equation (PBE)^68^. The parameters used for the calculations were the CHARMM parameters; the scale was assigned a resolution of 2 grids/Å; the perfil was set at 70; the dielectric constant for water and protein were set at 80 and 2, respectively. The salt concentration was set to an ionic strength of I = 0.15 M. Information regarding the parameters is available in the DelPhi manual (http://compbio.clemson.edu/downloadDir/delphi/delphi_manual.pdf). The distribution of the electrostatic potential was visualized using VMD.

Electrostatic forces for each of the MTBD-E-hook complexes were calculated using DelPhiForce^69^. To isolate the role of E-hooks on MTBD binding, only the E-hooks were charged and the corresponding electrostatic force exerted on the MTBD was calculated. The parameters used for the force calculation were the same as the ones for the electrostatic potential calculations.

## Electronic supplementary material

Supplementary information

Video 1

Video 2

Video 3
