# Supplementary material for: E-hooks provide guidance and a soft landing for the microtubule binding domain of dynein
Source: Sci Rep. 2018 Sep 5;8:13266. doi: 10.1038/s41598-018-31480-9 (PMC6125590; doi:10.1038/s41598-018-31480-9)
Supplement: Supplementary file 1 — Supplementary information [file 41598_2018_31480_MOESM1_ESM.pdf]

Supplementary material

E-hooks provide guidance and a soft landing for the microtubule binding domain of dynein

Nayere Tajielyato, Lin Li, Yunhui Peng, Joshua Alper, Emil Alexov

Methods

A 3D structure comprised of two α-tubulin β-tubulin dimers and a MTBD from cytoplasmic dynein was built as described below. Two dimers were used to account for the possibility that E-hooks from neighboring tubulins may interact with the MTBD (Fig. 1). First, the high affinity configuration of a mouse dynein MTBD-tubulin complex from the Protein Data Bank (PDB) [1] (course code: 3J1T.pdb [2]) provided a template for the binding of the MTBD to tubulin dimer. However, the structure of the tubulin dimer in 3J1T is of low resolution (9.7 Å). Thus, the model was improved by replacing this low resolution tubulin dimer structure with a higher resolution (3.5 Å) cow tubulin dimer (PDB ID 1JFF [3]). The GTP/ GDP and Mg are far away from binding interface of tubulins and MTBD and where not included in the simulations. Thus, not including these in the simulations is not important for the phenomena being investigated, namely the role of E-hooks on MTBD binding. The last step was to build a microtubule segment made of two α-β-tubulin dimers while preserving the binding mode of the MTBD. Then, the rotation and translation matrix of a microtubule structure (PDB ID 3J2U [4]) was used to generate a two-dimer microtubule segment. Several microtubule segment models were tested by adding tubulin dimers both laterally and longitudinally. However, no, or only a few, contacts between the E-hooks on these additional dimers and the MTBD were observed. Therefore, the rest of the modeling was done with the segment made of two dimers, as shown in Fig. 1.

Molecular dynamics (MD) simulations

The MD simulations were done with NAMD [5]. First, a 10,000-step energy minimization was performed for each structure using the CHARMM force field [6] and Generalized Born (GB) [7] implicit solvent model. The temperature was set at 300 K and the ion concentration was set at 0.15 M in the simulations. The cutoff used for calculating Born radius was set at 12.0 Å. The parameter, scale 1-4, is used to exclude all 1-3 bonded pairs from non-bonded interaction. Since the two tubulin dimers just represent a small segment of the entire microtubule, their conformations may exhibit unrealistically large changes during the simulation due to the lack of the constraining effects of neighboring tubulins. Therefore, harmonic constraints were applied to all tubulin residues, except for the E-hooks residues using a harmonic constraint energy function with a constraint scaling factor of 1.0. Similarly, the MTBD is just a part of the entire dynein structure and the construct that was crystalized was truncated in within the coiled-coil stalk domain (source code: 3J1T.pdb [2]). Thus, following our previous work [8], the three residues at the top of these truncated helices were constrained as well. The calculations are performed on Palmetto supercomputing center (https://www.palmetto.clemson.edu/). Each MD simulation takes 8 nodes and each node contains16 CPUs for both (i) α-tubulin β-tubulin dimers without E-hook and MTBD (ii) α-tubulin β-tubulin dimers with E-hook and MTBD. The average time of each MD simulation is 110 hours for 20 ns. In the calculations, the CPU type is Intel Xeon E5-2665.

(a)

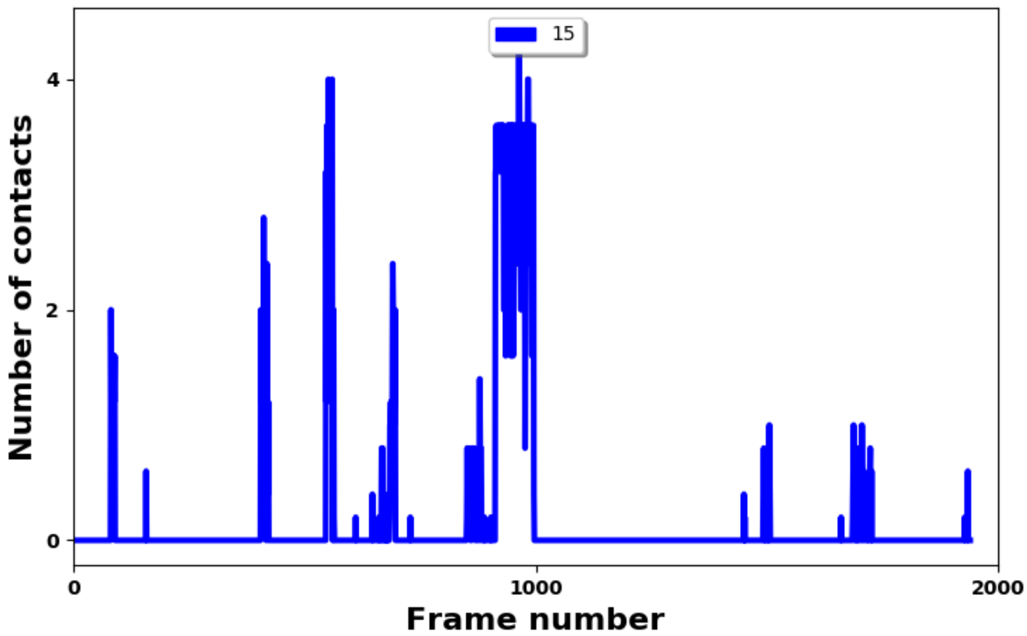

(b)

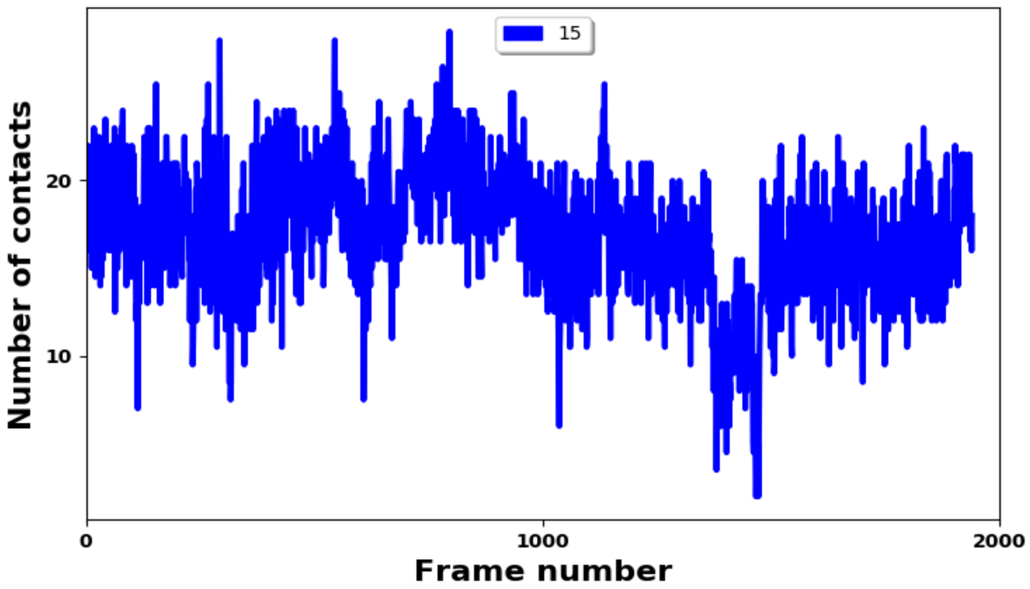

Fig. S1. Result for the extra run for 15 distance (a) chain B and (b) chain D

(a)

E-hook residues in chain B	MTBD in original position	MTBD in 5A°	MTBD in 15A°	MTBD in 25A°	MTBD in 35A°	MTBD in 45A°
ALA438	7	0	0	0	0	0
ASP439	1795	0	2	0	0	0
GLU440	31	6	16	0	0	0
GLN441	885	0	1	0	0	0
GLY442	85	0	0	0	0	0
GLU443	1132	22	0	0	0	0
PHE444	1264	1	0	1	0	0
GLU445	492	0	41	0	0	0
GLU446	147	11	39	0	0	0
GLU447	1090	1	12	1	0	0
GLY448	1	0	6	0	0	0
GLU449	44	6	12	0	0	0
GLU450	40	87	48	5	0	0
ASP451	0	0	2	31	55	1
GLU452	4	3	19	450	271	3
ALA453	2	0	54	132	186	1

(b)

E-hook residues in chain F	MTBD in original position	MTBD in 5A°	MTBD in 15A°	MTBD in 25A°
THR437	1	0	0	0
GLU440	30	21	0	0
GLN441	2	86	0	0
GLU443	0	2	0	0
PHE444	9	78	0	0
GLU445	60	2	0	0
GLU446	687	3	0	0
GLU447	346	46	0	0
GLY448	50	33	0	0
GLU449	163	165	0	0
GLU450	116	21	0	2
ASP451	138	74	0	21
GLU452	86	150	4	30
ALA453	88	33	0	7

Table S1. The total number of contacts between MTBD and the corresponding E-hook residues. (a) E-hook B residues and (b) E-hook F.

(a)

MTBD residues interacting with E-hook B	MTBD in origin position	MTBD in 5A°	MTBD in 15A°	MTBD in 25A°	MTBD in 35A°	MTBD in 45A°
LYS3295	47	0	0	0	0	0
SER3296	30	0	0	0	0	0
ILE3297	5	0	0	0	0	0
LYS3298	2558	49	53	2	0	0
LYS3299	610	0	113	21	92	0
GLN3300	124	0	47	0	0	0
HSE3301	64	0	0	1	0	0
ARG3306	0	0	0	0	417	0
SER3307	0	0	1	1	0	2
MET3308	0	0	0	7	0	3
ALA3309	0	0	0	9	0	0
ASN3310	0	0	0	19	0	0
PRO3312	0	0	0	5	0	0
ALA3313	0	0	0	12	0	0
TYR3377	2	3	0	0	0	0
ASN3381	61	0	0	0	0	0
ARG3382	2287	82	37	538	0	0
ALA3383	291	0	0	0	0	0
SER3384	208	0	0	0	0	0
LEU3385	563	0	0	0	0	0
ALA3386	146	0	0	0	0	0
CYS3387	7	0	0	0	0	0
GLY3388	2	0	0	0	0	0
LYS3392	12	3	0	0	0	0

(b)

MTBD residues interacting with E-hook C	MTBD in origin position	MTBD in 5A°	MTBD in 15A°	MTBD in 25A°
LYS3295	4	0	0	0
LEU3324	6	26	0	0
LEU3325	4	1	0	0
LEU3326	1	0	0	0
GLY3327	2	34	0	0
GLU3328	0	32	0	0
SER3329	27	70	0	2
ILE3339	8	0	0	0
ARG3342	907	0	0	0
GLU3343	1	0	0	0
ASN3344	161	0	0	0
PHE3345	23	0	0	0
PRO3347	16	0	0	0
THR3348	118	1	0	0

ASN3351	6	2	0	0
PHE3352	31	0	0	0
SER3353	21	0	0	0
SER3358	5	32	0	0
ALA3360	3	27	0	0
ILE3361	4	51	0	0
GLU3363	0	2	0	0
LYS3364	375	325	6	51
LYS3367	29	105	0	6
TYR3369	4	0	0	0
TYR3400	3	0	0	0
LEU3404	19	0	0	0

Table S2. The total number of contacts between the corresponding E-hook and MTBD residues. (a) MTBD residues interacting with E-hook B; (b) with E-hook F.

Helix number	MTBD residue numbers	Average RMSD(Å)	Average number of contacts
**H1**	3299-3305	1.67	3386
**H2**	3313-3326	1.33	13
**H3**	3332-3341	1.68	942
**H4**	3344-3351	1.52	356
**H5**	3358-3371	1.62	441
**H6**	3377-3383	1.91	2849

Table S3. Six helices of MTBD in bound state along with the average conformational changes and average contact numbers with the E-hooks.

a) MTBD-microtubule distance = 0Å

Free-E-hook▼	**Cluster 1 (25.24 %) N_c = 2183**	**Cluster 2 (20 .27%) N_c= 1830**	**Cluster 3 (10 %) N_c= 934**	**Cluster 4 (7 6.89%) | N_c= 348**	**Cluster 5 (6 5.99%) N_c = 307**
**Cluster 1 (33 .33%)**	4.52	1.99	**1.53**	**2.06**	3.02
**Cluster 2 (19.48%)**	5.62	4.49	5.12	4.32	5.30
**Cluster 3 (13 .22%)**	2.49	**1.54**	2.55	2.62	**1.40**
**Cluster 4 (11.5612 %)**	3.44	3.51	4.73	4.12	3.54
**Cluster 5 (7.29%)**	**1.29**	2.71	4.07	3.8	1.89

b) MTBD-microtubule distance = 5Å

Free-E-hook▼	**Cluster 1 (29 .48%) N_c =3**	**Cluster 2 (11 0.77%) N_c =0**	**Cluster 3 (9.9810 %) N_c = 5**	**Cluster 4 (8.959 %) N_c =0**	**Cluster 5 (5.37%) N_c =0**
**Cluster 1 (33.33%)**	**0.76**	2.48	2.28	6.22	2.13
**Cluster 2 (19.48%)**	4.53	3.52	5.25	5.22	3.73
**Cluster 3 (13.22%)**	2.12	2.42	**1.22**	4.69	2.01
**Cluster 4 (11.56%)**	4.23	2.79	3.93	3.43	2.91
**Cluster (7.29%)**	3.66	3.06	2.31	3.38	2.93

c) MTBD-microtubule distance = 15Å

Free-E-hook▼	**Cluster1 (14.51%) N_c =6**	**Cluster2 (10.29%) N_c =2**	**Cluster3 (9.81%) N_c =1**	**Cluster4 (6.56%) N_c =30**	**Cluster5 (5.32%) N_c =0**
**Cluster 1 (33.33%)**	2.87	5.28	3.98	2.65	2.17
**Cluster 2 (19.48%)**	3.56	4.01	2.42	4.69	4.01
**Cluster 3 (13.22%)**	2.15	3.94	3.67	**1.43**	2.34
**Cluster 4 (11.56%)**	**2.17**	**2.12**	**1.86**	3.22	3.12
**Cluster 5 (7.29%)**	2.42	2.85	3.6	1.96	3.26

d) MTBD-microtubule distance = 25Å

Free-E-hook▼	**Cluster1 (20.53%)**	**Cluster2 (16.30%)**	**Cluster3 (8.83%)**	**Cluster4 (8.65%)**	**Cluster5 (8.16%)**

	Nc =546	Nc =0	Nc =0	Nc =0	Nc =0
**Cluster 1 (33.33%)**	**1.97**	3.56	2.3	4.59	4.44
**Cluster 2 (19.48%)**	5.21	2.95	3.44	2.73	4.58
**Cluster 3 (13.22%)**	2.58	2.96	2.83	4.28	2.67
**Cluster 4 (11.56%)**	4.76	1.95	3.21	3.07	2.35
**Cluster 5 (7.29%)**	3.85	3	3.76	4.12	1.59

e) MTBD-microtubule distance = 35Å

**Free-E-hook▼**	**Cluster1 (21.11%) N_c =1**	**Cluster2 (15.44%) N_c =491**	**Cluster3 (8.95%) N_c =0**	**Cluster4 (8.52%) N_c =0**	**Cluster5 (6.23%) N_c =0**
**Cluster 1 (33.33%)**	**0.79**	3.82	4.74	2.97	3.03
**Cluster 2 (19.48%)**	4.52	5.5	2.36	3.7	2.12
**Cluster 3 (13.22%)**	1.98	1.9	4.21	4.47	3.57
**Cluster 4 (11.56%)**	4.07	3.68	1.87	4.9	2.59
**Cluster 5 (7.29%)**	3.46	**1.43**	3.81	5.54	4.08

f) MTBD-microtubule distance = 45Å

**Free-E-hook▼**	**Cluster1 (16.90%) N_c =2**	**Cluster2 (14.39%) N_c =0**	**Cluster3 (10.80%) N_c =0**	**Cluster4 (7.11%) N_c =0**	**Cluster5 (6.39%) N_c =0**
**Cluster 1 (33.33%)**	3.86	3.45	4.71	2.84	3.66
**Cluster 2 (19.48%)**	6.69	4.73	2.09	5.4	3.44
**Cluster 3 (13.22%)**	**3.14**	2.06	4.41	1.47	2.68
**Cluster 4 (11.56%)**	5.63	2.87	2.05	4.01	1.69
**Cluster 5 (7.29%)**	3.71	1.75	4.16	2.17	2.44

g) MTBD-microtubule distance = 55Å

**Free-E-hook▼**	**Cluster1 (40.63%) N_c =0**	**Cluster2 (30.10%) N_c =0**	**Cluster3 (5.79%) N_c =0**	**Cluster4 (5.41%) N_c =0**	**Cluster5 (5.13%) N_c =0**
**Cluster 1**	0.69	3.65	2.38	1.62	4.21

**(33.33%)**					
**Cluster 2 (19.48%)**	4.45	5.67	5.57	3.24	4.87
**Cluster 3 (13.22%)**	2.11	1.83	1.88	2.5	2.29
**Cluster 4 (11.56%)**	4.14	3.91	4.55	3.34	2.72
**Cluster 5 (7.29%)**	3.67	1.57	2.92	3.75	1.1

Table S4. Clustering analysis of the conformational states for E-hook B. Pairwise comparison of the first five most populated conformational clusters of E-hook B isolated from the MTBD (rows) with the first five most populated conformational clusters of E-hook B in the presence of the MTBD (columns). The MTBD-microtubule distances vary for each sub-table, with distances of 0, 5, 15, 25, 35, 45, and 55 Å corresponding to sub-table a-g, respectively. The population occupancy, which is the percentage of total snapshots from all 3 runs with the E-hook taking the confirmation of the respective cluster, is shown in parentheses. The number of MTBD-E-hook B contacts averaged over the 3 runs, N_c, is shown for each cluster. The RMSD between a representative of a cluster in free state (rows) and states with the MTBD bound (sub-table a) or situated away from microtubule (sub-tables b-g) is shown in Å. For example, the RMSD of the heavy atoms of the representative of the first free cluster (Cluster 1) and the representative of the first cluster in the bound state (Cluster 1) is 4.52 Å (first entry in sub-table a). For clusters having non-zero contacts, the smallest RMSD with respect to the free clusters is shown in bold.

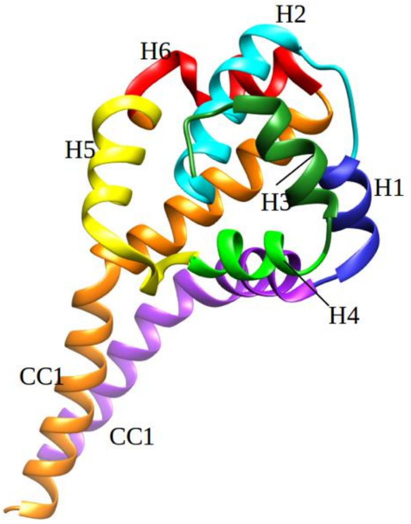

Fig. S2. MTBD with helices labeled.

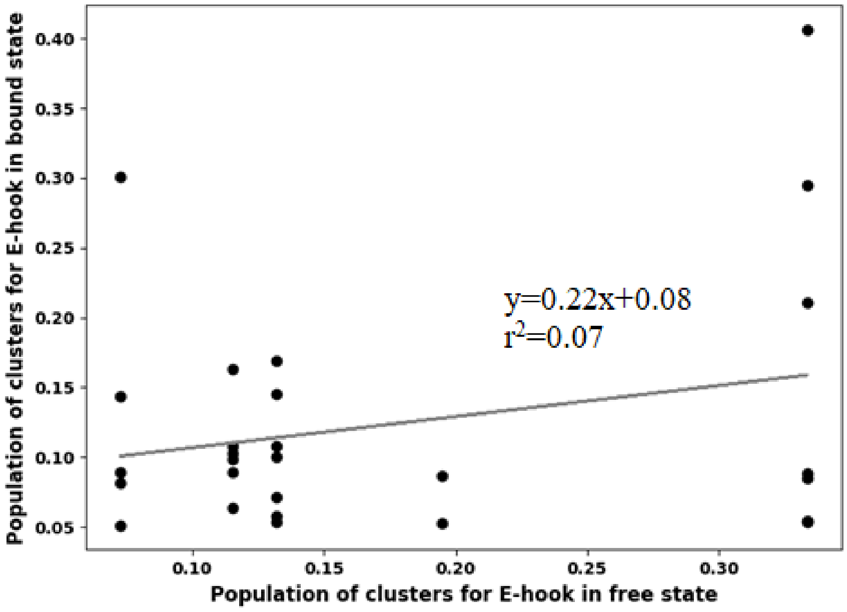

Fig. S3. The correspondence between the free clusters and clusters in bound state for clusters making no contacts.

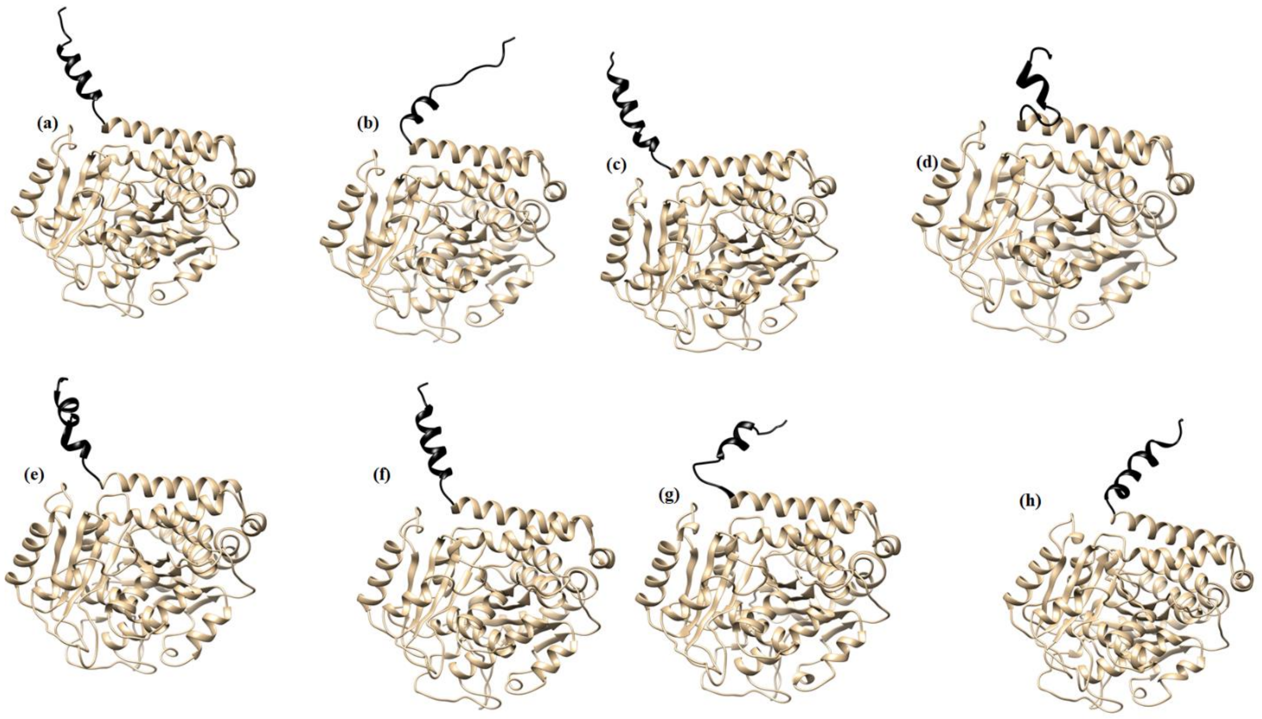

Fig. S4. Representative structures of the most populated cluster of E-hook B (shown in black color). Panel (a) presents the representative structure of E-hook B in free state and panel (b-h) are for E-hooks B in complex of MTBD at distances of 0, 5, 15, 25, 35, 45, and 55 Å from microtubule, respectively.

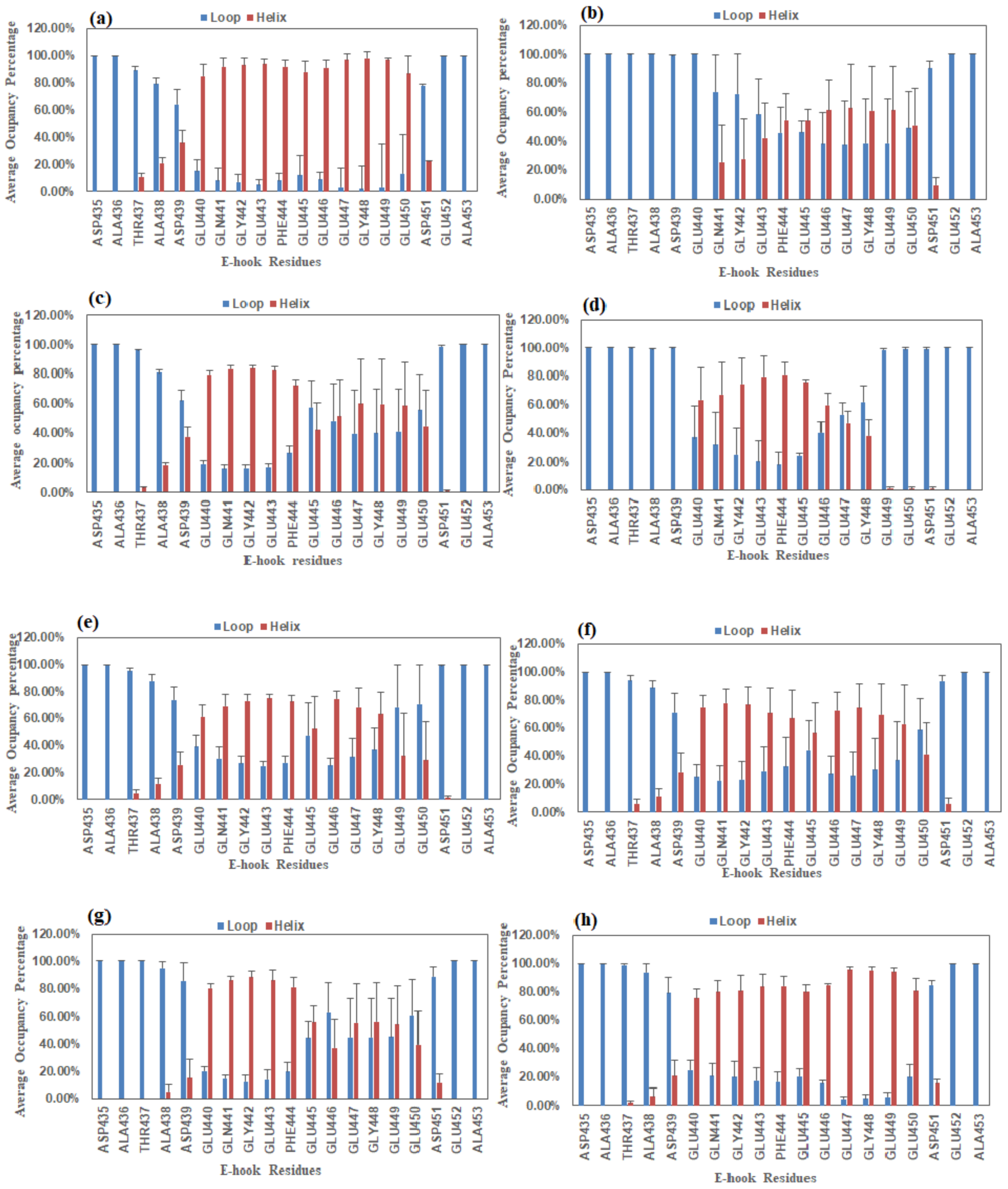

Fig. S5. The average percentage of secondary structural elements of E-hook B over 3 runs (total of 6000 snapshots). Blue and red bins are the percentage of structural elements of coil/bend/turns and helices occupying each residue of E-hook B, respectively. Panel(a) represents the secondary structure of E-hook B in free state and panels (b-h) are for E-hook B in complex with the MTBD at of 0, 5, 15, 25, 35, 45, and 55 Å from microtubule, respectively. It can be seen that helical content (especially in the middle of E-hook) is high in free state, while dramatically decreases at distances with many MTBD-E-Hook contacts. At very large distances (panel h), the helical content is similar to the helical content in free state (panel a).

To investigate the maximal contribution of individual amino acids of E-Hooks on MTBD binding free energy, we selected snap shots from 6,000 production runs at MTBD being in bound position. For each amino acid listed below, we selected only one frame at which this particular amino acid makes contact with MTBD. Using this frame, we carried MMGB/SA calculations (see details in the Table S5 below). The complex was modeled as MTBD with the particular E-Hook amino acid bound. The energy of the MTBD and separated E-Hook amino acid were calculated using the structures taken from the complex. This protocol estimates the maximal contribution of a particular amino acid to the MTBD binding since it takes into consideration only the best binding mode. Thus, if a given amino acids is mutated to Ala, the experimental effect is expected to be smaller than the energies listed in Table S5.

(a) Case of residues in E-Hook of chain B

E-Hook amino acid	Energy of the complex	Energy of MTBD alone	Energy of E-Hook residue	ΔG= $G_{complex}-G_{MTBD}-G_{E\text{-}hook\ res}$ (kcal/mol)
Glu447	-5479.0549	-5275.1041	-200.7953	-3.1555
Glu443	-5480.1081	-5273.6498	-202.0156	-4.4427
Asp439	-5514.1814	-5274.4868	-235.1006	-4.594
Glu452	-5477.9456	-5274.0615	-202.0206	-1.8635

(b) Case of residues in E-Hook of chain F

E-Hook amino acid	Energy of the complex	Energy of MTBD alone	Energy of E-Hook residue	ΔG= $G_{complex}-G_{MTBD}-G_{E\text{-}hook\ res}$ (kcal/mol)
Glu447	-5445.8397	-5243.0635	-200.795	-1.9812
Glu446	-5444.5661	-5242.3446	-200.0554	-2.1661
Glu452	-5444.5036	-5242.655	-200.7744	-1.0742

Table S5. MMGB/SA calculations of the binding free energy of selected residues and MTBD at bound position. Negative ΔG indicates that the amino acid contributes favorable to the MTBD binding.

References:

1 Berman, H., Henrick, K. & Nakamura, H. Announcing the worldwide Protein Data Bank. *Nature Structural Biology* **10**, 980-980, doi:10.1038/nsb1203-980 (2003).

2 Redwine, W. B. *et al.* Structural Basis for Microtubule Binding and Release by Dynein. *Science* **337**, 1532-1536, doi:10.1126/science.1224151 (2012).

3 Lowe, J., Li, H., Downing, K. H. & Nogales, E. Refined structure of alpha beta-tubulin at 3.5 A resolution. *J Mol Biol* **313**, 1045-1057, doi:10.1006/jmbi.2001.5077 (2001).

4 Asenjo, A. B. *et al.* Structural model for tubulin recognition and deformation by kinesin-13 microtubule depolymerases. *Cell Rep* **3**, 759-768, doi:10.1016/j.celrep.2013.01.030 (2013).

5 Nelson, M. T. *et al.* NAMD: a Parallel, Object-Oriented Molecular Dynamics Program. *The International Journal of Supercomputer Applications and High Performance Computing* **10**, 251-268, doi:10.1177/109434209601000401 (2016).

6 Vanommeslaeghe, K. *et al.* CHARMM general force field: A force field for drug-like molecules compatible with the CHARMM all-atom additive biological force fields. *J Comput Chem* **31**, 671-690, doi:10.1002/jcc.21367 (2010).

7 Onufriev, A., Case, D. A. & Bashford, D. Effective Born radii in the generalized Born approximation: the importance of being perfect. *J Comput Chem* **23**, 1297-1304, doi:10.1002/jcc.10126 (2002).

8 Li, L., Alper, J. & Alexov, E. Cytoplasmic dynein binding, run length, and velocity are guided by long-range electrostatic interactions. *Scientific Reports* **6**, doi:10.1038/srep31523 (2016).
